# Pseudoaneurysm Versus Chronic Expanding Hematoma on MRI: Hematoma-like Lesions with Distinct Therapeutic Strategies

**DOI:** 10.3390/biomedicines13112834

**Published:** 2025-11-20

**Authors:** Seul Ki Lee, Jun-Ho Kim, Jee-Young Kim

**Affiliations:** 1 Department of Radiology, St. Vincent’s Hospital, College of Medicine, The Catholic University of Korea, Seoul 06591, Republic of Korea; 2 Department of Orthopedic Surgery, Hallym University Sacred Heart Hospital, Hallym University, 22, Gwanpyeong-ro 170beon-gil, Dongan-gu, Anyang-si 13496, Republic of Korea

**Keywords:** pseudoaneurysm, chronic expanding hematoma, magnetic resonance imaging, hematoma

## Abstract

**Background/Objectives:** Pseudoaneurysm and chronic expanding hematoma (CEH) are uncommon soft tissue lesions that can mimic hematoma or hemorrhagic tumors on magnetic resonance imaging (MRI). Because treatment strategies differ, accurate differentiation is important. This study aimed to compare MRI characteristics of pseudoaneurysm and CEH and identify distinguishing imaging features. **Methods:** We retrospectively reviewed 12 patients diagnosed between June 2010 and June 2023 with pseudoaneurysm (n = 6) or CEH (n = 6). Patient demographics, lesion depth, and size were compared. MRI features were evaluated for morphology, internal characteristics, pulsatile artifact, and involvement of adjacent structures. **Results:** Pseudoaneurysms were consistently located in the muscle layer, whereas CEHs were predominantly found in the subcutaneous fat layer (83.3%, *p* = 0.015). CEHs were significantly larger than pseudoaneurysms (13.5 ± 3.9 cm vs. 6.1 ± 3.3 cm, *p* = 0.005). Pseudoaneurysm more frequently exhibited ovoid morphology (100%), central flow void on T1WI and T2WI (100%), inner peripheral high SI on T1WI (83.3%), and neurovascular bundle involvement (100%) (all *p* < 0.05), while CEHs demonstrated multilobular morphology (100%) and internal septations (83.3%) (*p* < 0.05). **Conclusions:** Lesion location, size, morphology, central flow void, inner peripheral high T1 signal, septation, and neurovascular involvement enables reliable MRI differentiation between pseudoaneurysm and CEH, guiding accurate diagnosis and guiding appropriate management.

## 1. Introduction

When a soft tissue mass is suspected, magnetic resonance imaging (MRI) is generally prioritized in the diagnostic workflow because of its superior soft tissue contrast. MRI not only delineates lesion morphology and extent but also provides detailed information on internal composition [1,2]. It is particularly sensitive for detecting hematomas and hemorrhagic components owing to the characteristic signal intensity (SI) changes in blood products such as methemoglobin and hemosiderin across sequences. [3]. This is clinically relevant since several soft tissue tumors, including synovial sarcoma, angiosarcoma, and high-grade undifferentiated pleomorphic sarcoma, are known for their frequent hemorrhagic changes [4]. Moreover, recent studies have increasingly emphasized the role of advanced MRI techniques—such as diffusion-weighted and dynamic contrast-enhanced imaging—to distinguish hematomas from hemorrhagic soft tissue tumors and avoid unnecessary biopsy in equivocal cases [5,6,7,8].

Although most previous research has focused on differentiating hematomas from hemorrhagic tumors, few studies have systematically compared pseudoaneurysm and chronic expanding hematoma (CEH), and most existing literature consists of isolated case reports or small single-center series [9,10,11,12,13,14]. As a result, standardized MRI criteria to distinguish these two lesions remain poorly defined, and they can appear similar on MRI despite having distinct etiologies and management pathways [11,12]. Pseudoaneurysm represents a focal breach of the vessel wall and typically requires urgent endovascular or surgical repair to prevent rupture and life-threatening hemorrhage [15,16]. In contrast, CEH is a long-standing encapsulated blood collection that may slowly enlarge, and surgical excision is often considered if it continues to expand or becomes symptomatic [17]. Accurate distinction between these conditions is essential, as misclassification can result in inappropriate or delayed treatment, whereas simple hematomas usually resolve spontaneously and can often be managed conservatively with observation [18].

In this study, we aim to investigate how pseudoaneurysm and CEH can be distinguished when a lesion resembling a chronic hematoma is encountered on MRI. We hypothesize that these entities differ in characteristic imaging features—including typical anatomical locations, relationship to the neurovascular bundle, central and peripheral SI, and enhancement patterns. Establishing such diagnostic criteria could improve diagnostic confidence and guide appropriate treatment decisions, ultimately improving patient outcomes.

## 2. Materials and Methods

### 2.1. Study Population

This retrospective study was approved by the institutional review board of our institution, and the requirement for informed consent was waived due to the retrospective nature of the study.

We reviewed medical records and imaging archives between June 2010 and June 2023 at a single tertiary referral center. Inclusion criteria were as follows: (1) patients with histopathologically or clinically confirmed pseudoaneurysm or CEH and (2) available MRI. Exclusion criteria included: (1) acute traumatic hematoma (n = 5) and (2) incomplete or poor-quality MRI data (n = 2). A total of 12 patients were finally included—pseudoaneurysm (n = 6) and CEH (n = 6). The diagnosis of pseudoaneurysm was confirmed by surgical exploration or angiography, while CEH was diagnosed based on surgical resection and histology. Patient demographics such as age and sex were recorded.

### 2.2. MRI Acquisition

All MRI examinations were performed with 1.5-T (Ingenia, Philips Healthcare, Best, The Netherlands) or 3.0-T scanners (Magnetom Verio or Magnetom Vida, Siemens Healthineers, Erlangen, Germany). The imaging protocol included the following sequences: spin-echo T1-weighted image (T1WI, TR/TE range, 370–693/10-19 in 1.5-T, 623/11 in 3.0-T), spin-echo T2-weighted image (T2WI) with and without fat suppression (TR/TE range: 1648–3280/80–100 in 1.5-T, 4000–6200/63–76 in 3.0-T), and contrast enhanced (CE) T1WI with fat suppression following intravenous administration of gadolinium-based contrast agent (0.1 mmol/kg). All images were acquired in axial, coronal, and sagittal planes. Slice thickness ranged from 3 to 5 mm, with interslice gaps of 0–0.5 mm. Standardized positioning and field-of-view parameters were maintained to ensure consistency across subjects.

### 2.3. Image Analysis

All images were independently reviewed by two musculoskeletal radiologists with 7 and 26 years of clinical experience of musculoskeletal imaging, respectively; both radiologists were blinded to the clinical and/or pathologic findings. Any discrepancies were resolved by consensus.

First, each case was evaluated for lesion location and maximum size. Lesion location was categorized as subcutaneous, fascial, or intramuscular layer based on the predominant site of origin [19,20]. Maximum lesion size was measured in centimeters on the plane showing the greatest extent [21].

**Figure 1:**
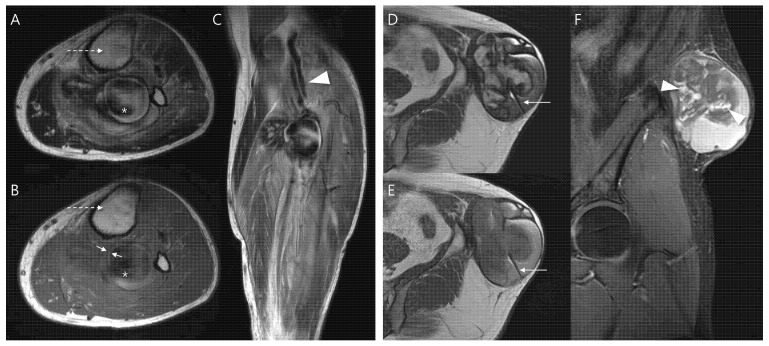
MRI findings of pseudoaneurysm (**A**–**C**) and chronic expanding hematoma (**D**–**F**). (**A**) Axial T2-weighted and (**B**) T1-weighted images show an intramuscular, ovoid mass with a central flow void (asterisk in (**A**,**B**), fast-flow blood), pulsatile artifact (dotted arrow in (**A**,**B**) and inner peripheral high SI on T1-weighted image (arrows in (**B**), mural thrombus). (**C**) Coronal T2-weighted image demonstrates neurovascular involvement (arrowhead). (**D**) Axial T2-weighted and (**E**) T1-weighted images reveal a multilobular subcutaneous mass with heterogeneous signal intensity with septations (arrow in (**D**,**E**)). (**F**) Coronal T1-weighted contrast-enhanced image demonstrates nodular enhancement (arrowheads, granulation tissue) within the mass.

Second, the following MRI features were assessed—including lesion morphology, internal characteristics, pulsatile artifact, and relationship to adjacent structures (Figure 1). Morphology was categorized as either ovoid or multilobular in contour. Internal characteristics related to hematoma were evaluated as follows: (1) outermost peripheral low SI on T1WI—indicating a fibrous pseudocapsule with hemosiderin or collagenous tissue [22,23]; (2) inner peripheral high SI on T1WI, located just inside the low-signal rim—reflecting mural thrombus with methemoglobin [11]; (3) central high SI on T1WI—consistent with subacute/mature methemoglobin or repetitive rebleeding deposits [24]; (4) central flow void on T1WI and T2WI, appearing as a distinct signal void or markedly hypointense core due to rapid intraluminal blood flow, observed on all sequences (but most conspicuous on T2WI due to strong flow-related dephasing)—indicative of fast-flow blood [25,26]; (5) septation—representing internal fibrous septa or loculations [5]; and (6) nodular enhancement—suggesting granulation tissue or neovascularization.

A pulsatile artifact (Figure 1) was defined as a motion-related imaging artifact on MRI that arises from the periodic pulsation of blood flow, typically appearing as ghosting of SI along the phase-encoding direction and was considered as an indirect marker of active vascular flow [27,28].

Last, neurovascular bundle involvement (Figure 1) was considered positive when displacement, encasement or infiltration of adjacent neurovascular structures was observed [29].

### 2.4. Statistical Analysis

All statistical analyses were performed using SPSS version 26.0 (IBM Corp., Armonk, NY, USA). Normality of continuous variables was assessed using the Shapiro–Wilk test. Student’s *t*-test was performed to compare ages and maximum lesion size between groups, and results were summarized as means and standard deviations (SD). Categorical variables were compared between groups using Fisher’s exact test. Interobserver agreements for imaging features were assessed using Cohen’s kappa (κ) statistics. The strength of agreement was interpreted according to the guidelines proposed by Landis and Koch [30]: values of κ < 0.00 indicated poor agreement, 0.00–0.20 slight, 0.21–0.40 fair, 0.41–0.60 moderate, 0.61–0.80 substantial, and 0.81–1.00 almost perfect agreement. A *p* value < 0.05 was considered statistically significant.

## 3. Results

### 3.1. Patient Characteristics

The mean age was 70.7 ± 10.6 years (age range, 53–83 years; 3 women and 3 men) in the pseudoaneurysm group (n = 6) and 66.3 ± 5.6 years (age range, 57–72 years; 2 women and 4 men) in the CEH group (n = 6). There was no significant difference in age (*p* = 0.403) or sex distribution (*p* = 1.000) between the two groups. Pseudoaneurysms were exclusively intramuscular (100%), whereas CEHs were predominantly subcutaneous layer (5/6, 83.3%, *p* = 0.015). CEHs were significantly larger than pseudoaneurysms, with a mean maximum diameter of 13.5 ± 3.9 cm compared to 6.1 ± 3.3 cm (*p* = 0.005). Detailed patient demographics and lesion characteristics are summarized in Table 1.

### 3.2. MRI Features Regarding Morphology, Internal Characteristics, Pulsatile Artifact, and Relationship to Adjacent Structures

Pseudoaneurysms consistently showed ovoid morphology (6/6, 100%) and central flow void on both T1WI and T2WI (6/6, 100%). Inner peripheral high SI on T1WI, suggestive of mural thrombus containing methemoglobin, was present in 5/6 (83.3%) cases. Neurovascular bundle involvement was observed in all pseudoaneurysms (6/6, 100%). In contrast, CEHs showed multilobular morphology in all cases (6/6, 100%) and frequent internal septations (5/6, 83.3%), reflecting fibrous bands within the hematoma cavity. Pseudoaneurysms and CEHs shared overlapping hematoma-like features, including outermost peripheral low SI and central high SI on T1WI, as well as occasional nodular enhancement. Although a pulsatile artifact was considered a potential marker of pseudoaneurysm, it was not consistently observed in pseudoaneurysm and did not reach statistical significance in differentiating between the two groups. The results are summarized in Table 2. Interobserver agreement was excellent across all MRI features, with κ values ranging from 0.74 to 1.00 (Table 3).

### 3.3. MRI-Based Diagnostic Flowchart for Differentiating Pseudoaneurysm and CEH

**Figure 2:**
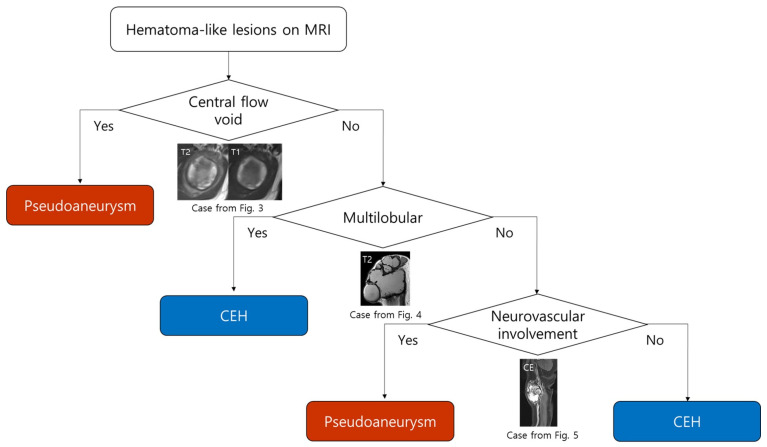
Diagnostic flowchart for differentiating pseudoaneurysm and CEH on MRI. The presence of a central flow void strongly suggests pseudoaneurysm. In the absence of central flow void, a multilobular morphology favors CEH. For non-multilobular lesions, evaluation of neurovascular involvement is helpful; lesions with neurovascular involvement are more suggestive of pseudoaneurysm, while those without involvement are consistent with CEH.

A diagnostic flowchart was constructed to differentiate pseudoaneurysm from CEH among hematoma-like lesions on MRI (Figure 2). The presence of a central flow void was considered the most reliable indicator and consistently classified the lesion as pseudoaneurysm. In the absence of central flow void, multilobular morphology strongly favored the diagnosis of CEH, reflecting its chronic remodeling process. For non-multilobular lesions, neurovascular bundle involvement was regarded as a key supportive feature of pseudoaneurysm, whereas its absence suggested CEH. This stepwise approach highlights the practical value of combining vascular-specific signs (central flow void and neurovascular involvement) with morphologic criteria (multilobular contour) to improve diagnostic confidence and reduce misclassification.

### 3.4. Application of the Diagnostic Flowchart: Representative Cases

**Figure 3:**
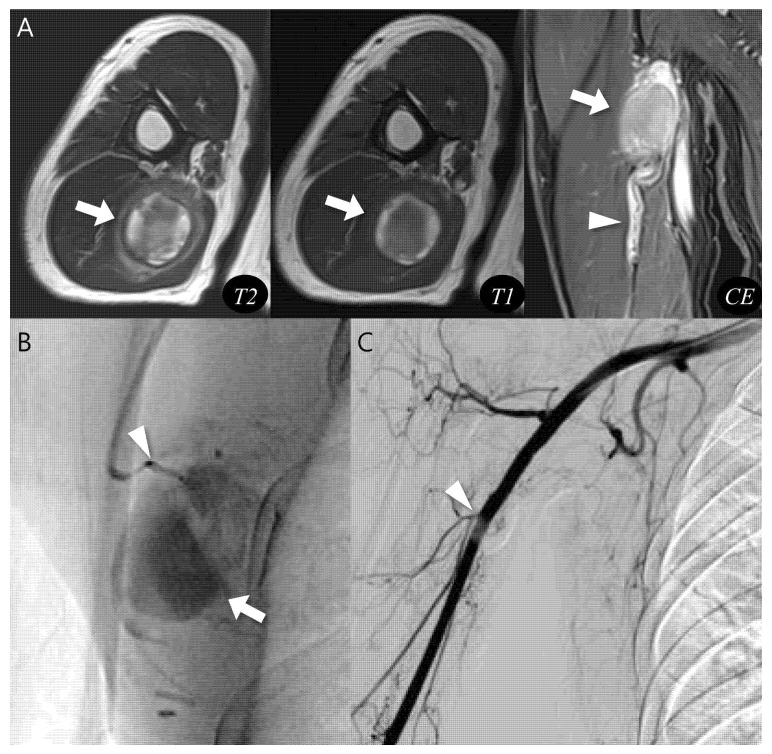
Diagnosis and treatment of pseudoaneurysm. (**A**) Axial T2-weighted, T1-weighted, and coronal T1-weighted contrast-enhanced MR images show an intramuscular, ovoid lesions (arrow) in the upper arm. The lesion demonstrates a central flow void with inner peripheral high SI on T1-weighted image (arrow, mural thrombus) with neurovascular involvement (arrowhead). (**B**) Digital subtraction angiography reveals a pseudoaneurysm sac (arrow) with a narrow neck arising from the brachial artery (arrowhead). (**C**) Post-embolization angiography demonstrates successful occlusion of the pseudoaneurysm neck (arrowhead).

**Figure 4:**
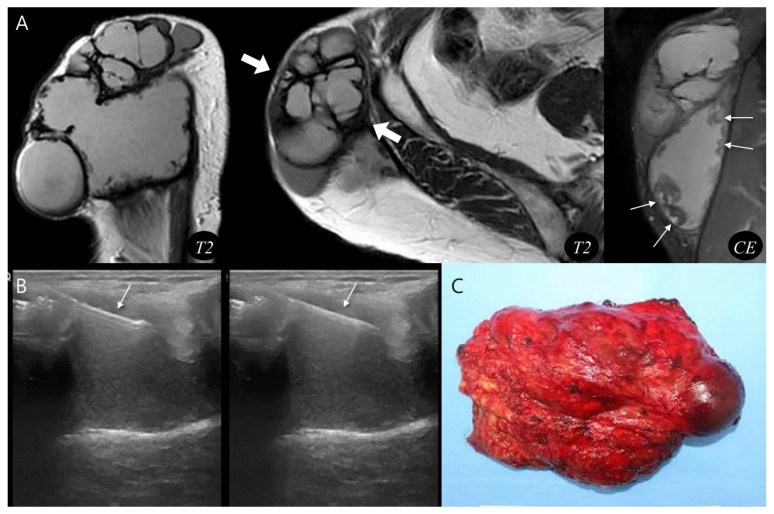
Diagnosis and treatment of chronic expanding hematoma. (**A**) Sagittal and axial T2-weighted MR images demonstrate a multilobular, subcutaneous mass (thick arrows) with internal septations. The lesion shows peripheral nodular enhancement (thin arrows, granulation tissue) on coronal T1-weighted contrast-enhanced MR image. (**B**) Ultrasound-guided biopsy was performed using a core needle (arrows), confirming the hematoma. (**C**) Gross photograph of the wide excision demonstrates chronic expanding hematoma.

Using the proposed diagnostic flowchart, we successfully applied this approach in representative cases. In a representative pseudoaneurysm case (Figure 3), the hematoma-like lesion demonstrated a central flow void with adjacent neurovascular bundle involvement on MRI, which allowed a confident diagnosis of pseudoaneurysm; the patient subsequently underwent successful endovascular embolization. In contrast, in a CEH case (Figure 4), the lesion exhibited a multilobular hematoma-like lesion with internal septations and lacked neurovascular bundle involvement, findings that favored the diagnosis of CEH and were later confirmed after wide surgical excision. These representative cases underscore the practical utility of the flowchart by illustrating how specific imaging features can be translated into accurate diagnostic decisions and directly guide appropriate therapeutic strategies.

**Figure 5:**
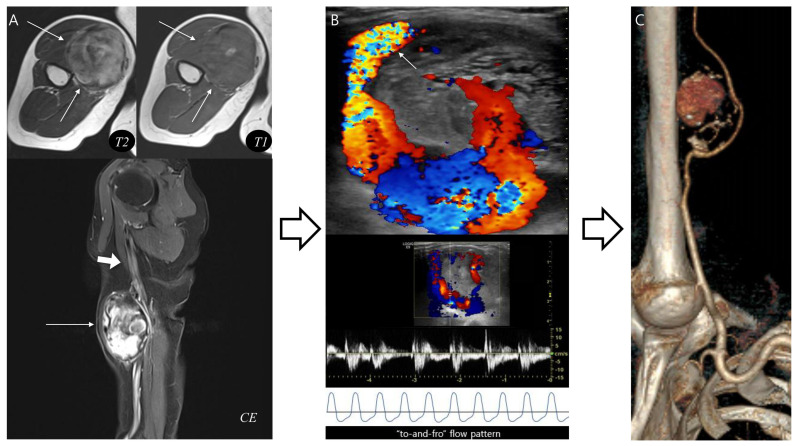
Pseudoaneurysm with neurovascular bundle involvement diagnosed without biopsy. (**A**) Axial T2-weighted and T1-weighted MR images demonstrate a hematoma-like lesions (thin arrows) with equivocal central flow void or absent inner peripheral high SI. However, neurovascular bundle involvement (thick arrow) in sagittal T1-weighted contrast-enhanced MR image raises suspicion for pseudoaneurysm. (**B**) Doppler ultrasonography reveals a characteristic “Yin-yang” sign with neck at the brachial artery (arrow), confirming a “to-and-fro” flow pattern. (**C**) CT angiography with 3D reconstruction demonstrates a pseudoaneurysm arising from the brachial artery, consistent with the MRI suspicion. This case highlights that recognition of distinct MRI features can guide appropriate diagnosis without unnecessary biopsy.

In the last pseudoaneurysm case (Figure 5), typical MRI findings, such as a central flow void was equivocal and inner peripheral high SI on T1WI was absent, making the diagnosis more challenging. Instead, the presence of neurovascular bundle involvement raised strong suspicion for pseudoaneurysm which was subsequently confirmed by complementary vascular imaging. This case demonstrates that the diagnostic flowchart retains its clinical value even in atypical presentations, underscoring the importance of integrating ancillary features such as neurovascular involvement to achieve accurate diagnosis and ensure appropriate treatment.

## 4. Discussion

This study investigated MRI features of pseudoaneurysms and CEHs, both of which frequently mimic hematoma-like lesions on imaging and can be easily misclassified, despite having markedly different clinical courses and management requirements. We identified significant differences in lesion location, morphology, internal characteristics, and neurovascular involvement. These imaging findings were subsequently incorporated into a diagnostic flowchart. Such a flowchart not only enhances diagnostic confidence but also provides a reproducible approach for differentiating these entities, thereby facilitating timely treatment selection and reducing the risk of unnecessary biopsy or delayed intervention.

In our study, pseudoaneurysms demonstrated several characteristic MRI features that distinguished them from CEHs. These included a consistently ovoid morphology, the presence of a central flow void on both T1WI and T2WI, frequent inner peripheral high SI on T1WI, and consistent involvement of the adjacent neurovascular bundle. These findings are in line with prior studies describing pseudoaneurysm as a lesion with a central flow void corresponding to actively circulating fast-flowing blood within the patent lumen. This intraluminal flow void is widely regarded as a hallmark of vascular lesions on spin-echo sequences, reflecting not only the turbulence but also the velocity and directionality of arterial blood, and its identification provides a reliable noninvasive marker of active vascular communication [31]. Frequent inner peripheral high SI in most pseudoaneurysm cases likely reflects mural thrombus with methemoglobin, which accumulates over time [32]. Earlier reports have also emphasized that neurovascular involvement is an important diagnostic clue, as pseudoaneurysms often originate directly from arterial injury or disruption. Because pseudoaneurysms arise from a focal breach in the arterial wall, they typically remain in continuity, explaining their frequent proximity to major neurovascular bundles and highlighting the importance of careful assessment of adjacent structures during MRI interpretation [33]. Although a pulsatile artifact has been suggested as a potential marker of pseudoaneurysm [34], it was not consistently observed across all cases. The variability in its detection may be attributed to differences in MRI acquisition parameters [35]. In deep or small pseudoaneurysm, flow-related motion can be partially suppressed [36]. Despite its limited sensitivity, the presence of a pulsatile artifact remains a supportive indicator of active blood flow and should prompt careful evaluation for possible pseudoaneurysm [37].

By contrast, CEHs were significantly larger in size and most commonly located in the subcutaneous fat layer. This is consistent with the concept that long-standing hematomas progressively expand due to repeated minor bleeding episodes into the lesion cavity and fibrous capsule formation [5,22,38]. The accumulation of hemosiderin and the gradual organization of fibrin lead to the development of a dense fibrous capsule, which not only stabilizes the lesion but also predisposes to recurrent bleeding from fragile neocapillaries formed within the capsule wall [23,39]. This vicious cycle of hemorrhage and encapsulation accounts for the characteristic slow but relentless enlargement of CEHs and their frequent occurrence in the superficial soft tissue compartments [17]. Morphologically, CEHs uniformly showed a multilobular contour with frequent internal septations, reflecting the presence of fibrous bands and organization within the hematoma cavity [23]. These findings align with earlier studies reporting multilobular or septated appearance as a hallmark of CEH, attributed to chronic remodeling and encapsulation processes rather than active vascular communication [22,40]. In particular, the presence of fibrous septa and multilobulated architecture underscores the chronicity of the lesion and distinguishes CEHs from pseudoaneurysm [12]. Furthermore, the recognition of these morphologic features is clinically meaningful, because CEHs can be misinterpreted as aggressive soft tissue neoplasms owing to their large size, heterogeneous signal intensity, and nodular or septal enhancement. This erroneous diagnosis can lead to unnecessary biopsy or overly aggressive treatment approaches [10]. Taken together, these features highlight the importance of recognizing CEH-specific imaging patterns to avoid misdiagnosis and to guide appropriate management [17].

Both pseudoaneurysms and CEHs demonstrate overlapping hematoma-like features, such as outermost peripheral low signal on T1WI and occasional nodular enhancement [23]. This overlap likely explains why these lesions are often misclassified as either simple hematomas or hemorrhagic tumors in clinical practice, a pitfall that can significantly delay appropriate treatment or lead to harmful interventions [10]. Our study proposed a diagnostic flowchart that can be readily applied in daily practice; while pseudoaneurysms require prompt endovascular or surgical repair to prevent rupture and life-threatening hemorrhage [15,16], CEHs are more appropriately managed with surgical excision when symptomatic or enlarging [17]. Misclassification could result in catastrophic hemorrhage if a pseudoaneurysm was mistaken for a benign hematoma and biopsied, or in unnecessary intervention if a CEH was misinterpreted as a vascular lesion [11]. Our proposed flowchart—beginning with the evaluation of central flow voids, followed by that of morphology and neurovascular involvement—could provide radiologists and clinicians with practical diagnostic clues that can improve patient safety and optimize therapeutic strategies. This highlights the robustness of the proposed algorithm, even in cases lacking classic findings. Although inner peripheral high SI on T1WI was statistically significant, it was excluded from the flowchart because central flow void was a more reliable discriminator and one pseudoaneurysm case lacked this finding.

The major strength of this study lies in the direct comparison of pseudoaneurysm and CEH, two rare hematoma-like lesions that are often difficult to distinguish on MRI. The study identified reproducible MRI parameters that are both diagnostically and clinically relevant. The proposed diagnostic flowchart offers a practical approach for clinical decision-making. Moreover, this study bridges radiologic interpretation with therapeutic relevance, providing a framework that can be readily applied in daily clinical practice.

Several limitations should be acknowledged. First, this was a retrospective study with a relatively small sample size (only 12 cases, 6 in each group). This might have limited the statistical power of subgroup analyses and limited the ability to detect subtle differences between entities, although the rarity of both conditions partly accounts for this constraint. Future multicenter studies with larger patient populations are necessary to validate the proposed diagnostic criteria. Second, although our study focused on pseudoaneurysm and CEH, other hematoma-like lesions such as hemorrhagic tumors were not included, which may restrict the broader applicability and external validity of the diagnostic flowchart in routine clinical practice. The proposed flowchart was specifically designed to differentiate pseudoaneurysm from CEH among hematoma-like lesions. Its application does not extend to other hemorrhagic or vascular mimickers such as hemorrhagic soft tissue tumors or arteriovenous malformation, which can share overlapping imaging features. When the diagnosis remains uncertain, additional imaging or pathologic confirmation should be considered to avoid misdiagnosis and ensure appropriate management. Future studies incorporating a wider spectrum of hematoma-mimicking lesions—including hemorrhagic sarcomas or vascular malformations—are warranted to strengthen the generalizability and clinical robustness of the present findings. Given the limitations of qualitative MRI assessment, we also plan to integrate quantitative approaches—including radiomics, histogram analysis, and machine learning—in future studies with larger cohorts. Such methods will likely enhance diagnostic accuracy and ensure greater reproducibility in differentiating pseudoaneurysm from CEH.

## 5. Conclusions

Pseudoaneurysm and CEH are rare but clinically important hematoma-like lesions that frequently mimic not only one another but also hemorrhagic soft tissue tumors on MRI, creating a substantial diagnostic challenge. Our study demonstrated that careful evaluation of lesion location, morphology, central flow void, inner peripheral high signal on T1WI, septation, and neurovascular bundle involvement allows reliable differentiation between the two entities. Notably, pseudoaneurysms consistently showed ovoid morphology (100%), central flow void (100%), and neurovascular involvement (100%), while CEHs uniformly demonstrated multilobular contours (100%) and frequent septations (83.3%). The innovation of this study lies in converting detailed MRI observations into a clinically applicable diagnostic algorithm, improving diagnostic confidence and preventing unnecessary biopsy or treatment delay. By focusing on key imaging features rather than purely descriptive interpretation, our approach provides radiologists with a structured method for distinguishing vascular and chronic encapsulated hematomatous lesions. Although the sample size was limited due to the rarity of these lesions, the diagnostic framework demonstrated consistent interpretability across cases.

## Figures and Tables

**Table 1:** Patient demographics and lesion characteristics between the two groups.

	Pseudoaneurysm	CEH	*p* Value
	(n = 6)	(n = 6)	
Age (years)	70.7 ± 10.6	66.3 ± 5.6	0.403
Sex			1.000
Male (%)	3 (50.0)	4 (66.7)	
Female (%)	3 (50.0)	2 (33.3)	
Depth			
Subcutaneous fat layer (%)	0 (0.0)	5 (83.3)	0.015
Fascial layer (%)	0 (0.0)	2 (33.3)	0.454
Muscle (%)	6 (100.0)	3 (50.0)	0.181
Maximum size (cm)	6.1 ± 3.3	13.5 ± 3.9	0.005

**Table 2:** Image analysis between the two groups.

	Pseudoaneurysm	CEH	*p* Value
	(n = 6)	(n = 6)	
Morphology			0.0024
Multilobular	0 (0.0%)	6 (100.0%)	
Ovoid	6 (100.0%)	0 (0.0%)	
Outermost peripheral low SI on T1WI	6 (100.0%)	6 (100.0%)	1.000
Inner peripheral high SI on T1WI	5 (83.3%)	0 (0.0%)	0.015
Central high SI on T1WI	4 (66.7%)	6 (100.0%)	0.454
Central flow void on T1,T2WI	6 (100.0%)	0 (0.0%)	0.002
Septation	0 (0.0%)	5 (83.3%)	0.015
Nodular enhancement	4 (66.7%)	5 (83.3%)	1.000
Pulsatile artifact	3 (50.0%)	0 (0.0%)	0.181
Neurovascular bundle involvement	6 (100.0%)	0 (0.0%)	0.002

**Table 3:** Interobserver agreements for MRI features.

MRI Feature	Kappa	95% CI	*p* Value
Morphology	0.80	0.45–1.00	0.003
Outermost peripheral low SI on T1WI	1.00	1.00–1.00	<0.001
Inner peripheral high SI on T1WI	0.78	0.42–1.00	0.004
Central high SI on T1WI	0.74	0.35–0.98	0.006
Central flow void on T1,T2WI	0.80	0.41–1.00	0.003
Septation	1.00	1.00–1.00	<0.001
Nodular enhancement	1.00	1.00–1.00	<0.001
Pulsatile artifact	1.00	1.00–1.00	<0.001
Neurovascular bundle involvement	1.00	1.00–1.00	<0.001

## Data Availability

Dataset available on request from the authors.
